# Supplementary material for: Identification of photoperiod-induced specific miRNAs in the adrenal glands of Sunite sheep (Ovis aries)
Source: Front Vet Sci. 2022 Jul 22;9:888207. doi: 10.3389/fvets.2022.888207 (PMC9354845; doi:10.3389/fvets.2022.888207)
Supplement: Supplementary file 1 [file Data_Sheet_1.ZIP › Supplementary file/miRDeep2 file.zip!/miRDeep2 file/Know_Structure/LP42A1/oar-mir-1197.pdf]

miRBase precursor : oar-mir-1197
Total read count : 61
oar-miR-1197-5p read count : 2
oar-miR-1197-3p read count : 52
remaining reads : 7

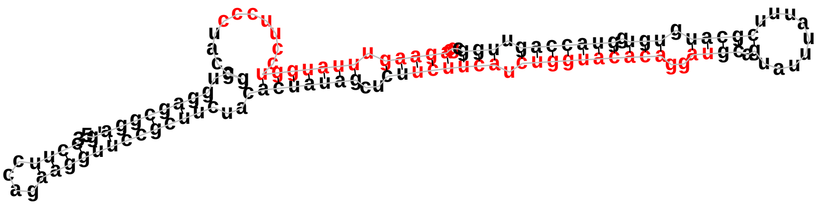

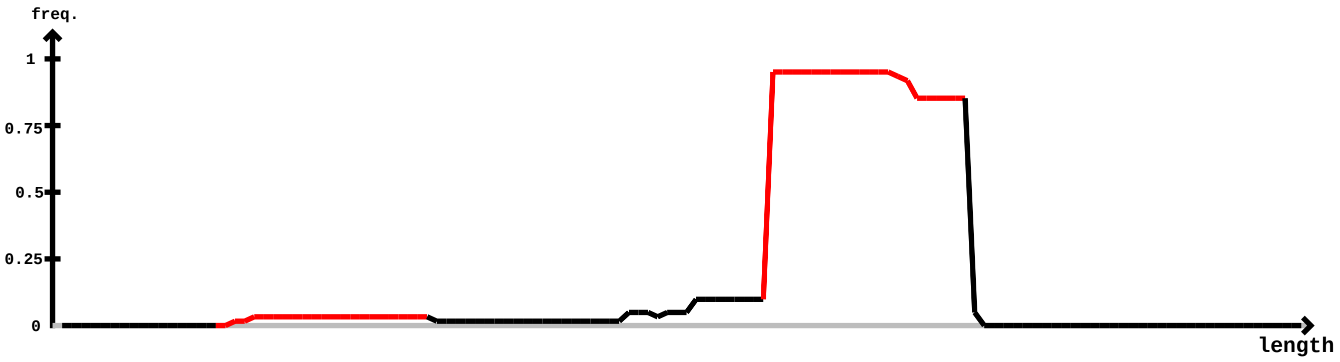


```
                                                                                       oar-miR-1197-3p
                 oar-miR-1197-5p
5'- gaggcgaggucggcaucccuuccugguauuugaagacgcgguugaccauggugugu acgcuuuauuuaugacguaggacacauggucuacuucuucucgauaucacaucuucgccuuggaagaccuucc   -3'    exp
    (((((((((..(...........(((((((.(((((...(((.(((((((.(((.(((((.........).))))..)))))))))).))))))))..))))))))..)))))))))((((....))))    reads      mm
    .................ccuuccugguauuugaagacg...........................................................................................    1          0
    ...................uuccugguauuugaagacg...........................................................................................    1          0
    ......................................cgguugaccauggugugu acgcu....................................................................    1          0
    ..........................................................gcuuuauuuaugacguaggacacaugguc..........................................    1          0
    ..........................................................gcuuuauuuaugacguaggacacauggucu.........................................    1          0
    ..............................................................uauuuaugacguaggacacauggucu.........................................    1          0
    .................................................................uuaugacguaggacacauggu...........................................    1          0
    .................................................................uuaugacguaggacacauggucu.........................................    2          0
    .........................................................................uaggacacauggucuacuucu...................................    49         0
    .........................................................................uaggacacauggucuacuucuu..................................    3          0
```
